# Epistatic and allelic interactions control expression of ribosomal RNA gene clusters in *Arabidopsis thaliana*

**DOI:** 10.1186/s13059-017-1209-z

**Published:** 2017-05-03

**Authors:** Fernando A. Rabanal, Terezie Mandáková, Luz M. Soto-Jiménez, Robert Greenhalgh, David L. Parrott, Stefan Lutzmayer, Joshua G. Steffen, Viktoria Nizhynska, Richard Mott, Martin A. Lysak, Richard M. Clark, Magnus Nordborg

**Affiliations:** 1 Gregor Mendel Institute (GMI), Austrian Academy of Sciences, Vienna Biocenter (VBC), Dr. Bohr-Gasse 3, 1030 Vienna, Austria; 2 Central European Institute of Technology (CEITEC), Masaryk University, Brno, Czech Republic; 3 Department of Biology, University of Utah, Salt Lake City, UT USA; 4 Department of Natural Sciences, Colby-Sawyer College, New London, NH USA; 5 Genetics Institute, University College London (UCL), Gower Street, London, WC1E 6BT UK; 6 Center for Cell and Genome Science, University of Utah, Salt Lake City, UT USA

**Keywords:** Ribosomes, rRNA genes, Transcription, Epistasis, Dominance

## Abstract

**Background:**

Ribosomal RNA (rRNA) accounts for the majority of the RNA in eukaryotic cells, and is encoded by hundreds to thousands of nearly identical gene copies, only a subset of which are active at any given time. In *Arabidopsis thaliana*, 45S rRNA genes are found in two large ribosomal DNA (rDNA) clusters and little is known about the contribution of each to the overall transcription pattern in the species.

**Results:**

By taking advantage of genome sequencing data from the 1001 Genomes Consortium, we characterize rRNA gene sequence variation within and among accessions. Notably, variation is not restricted to the pre-rRNA sequences removed during processing, but it is also present within the highly conserved ribosomal subunits. Through linkage mapping we assign these variants to a particular rDNA cluster unambiguously and use them as reporters of rDNA cluster-specific expression. We demonstrate that rDNA cluster-usage varies greatly among accessions and that rDNA cluster-specific expression and silencing is controlled via genetic interactions between entire rDNA cluster haplotypes (alleles).

**Conclusions:**

We show that rRNA gene cluster expression is controlled via complex epistatic and allelic interactions between rDNA haplotypes that apparently regulate the entire rRNA gene cluster. Furthermore, the sequence polymorphism we discovered implies that the pool of rRNA in a cell may be heterogeneous, which could have functional consequences.

**Electronic supplementary material:**

The online version of this article (doi:10.1186/s13059-017-1209-z) contains supplementary material, which is available to authorized users.

## Background

**Fig. 1:**
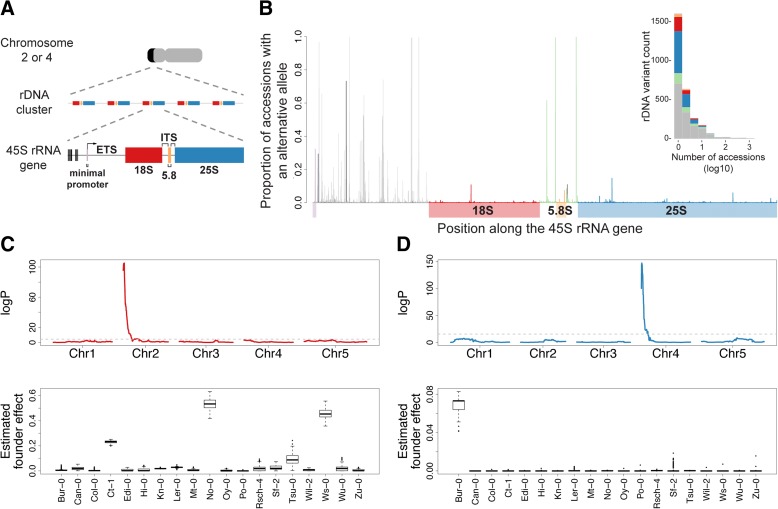
Identification and annotation of polymorphisms along the 45S rRNA gene. **a** *Schematic illustration* of the positioning of the rDNA clusters (in *black*) at the distal end region of chromosomes 2 or 4 (in *gray*) in *A. thaliana*, the head-to-tail tandem arrangement of the 45S rRNA genes, and the structure of each ~10 kb long 45S rRNA gene. **b** Proportion of accessions in the population (1138 individuals; see “Methods”) carrying a variable site (present in > 5% of copies within an individual) along the 45S rRNA gene. *Vertical lines* represent SNPs or deletions in the minimal promoter (*purple*), 5’ETS (*gray*), 18S (*red*), ITSs (*green*), 5.8S (*yellow*), and 25S (*blue*) regions along the 45S rRNA gene. *Black lines* depict insertions. *Inset* shows the distribution of rRNA gene variants shared across accessions, where the number of accessions is displayed in log10 scale. **c** Example of linkage mapping of the abundance of an 18S variable site (position 2882, T to C) estimated by DNA-sequence coverage in 393 individuals of the MAGIC population (*top*). Estimated founder accession effect by multiple imputation using R/happy [47, 90] at the major quantitative trait locus from the top panel (*bottom*). **d** Similar to (**c**), but for a 25S variable site (position 6661, G to A)

The central importance of ribosomal RNA (rRNA) genes for our understanding of biology cannot be overstated: they may well be evolutionarily the oldest genes [1–4]; they are the most highly expressed genes in any organism; and their expression is central to cellular growth [5]. In eukaryotes, the catalytic core of ribosomes consists of four RNA molecules: the 18S, 5.8S and 25S rRNAs, produced via a common 45S rRNA precursor; and the often separately encoded 5S rRNAs [6, 7]. Because of the requirement for large quantities of rRNA, hundreds to thousands of 45S rRNA genes are tandemly arrayed head-to-tail in large ribosomal DNA (rDNA) clusters that, when expressed, form nucleolus organizer regions (NORs) [8–11] (Fig. 1a).

Although rRNA accounts for the majority of the RNA in a eukaryotic cell, only a subset of the rRNA genes appear to be active at any given time: the others are silenced by repressive chromatin modifications [5, 12–14]. In interspecific hybrids, uniparental expression of rRNA genes due to an epigenetic phenomenon termed nucleolar dominance is often observed [13, 15–17]. The word “dominance” is used because one rDNA cluster apparently suppresses the activity of the other [18]. At the intraspecies level, many model organisms, such as humans, mice, zebrafish, wheat, and *Arabidopsis thaliana* have multiple rDNA clusters on different autosomes [19–23] and early cytogenetic studies have shown differential expression of such clusters in human cell types [24] and plants [25]. While attempts have been made to clone and sequence differentially expressed rRNA gene variants, it was not possible to assign them to a specific rDNA locus or cluster [26–28]. In humans, for instance, characterizing rDNA cluster-specific variation required rodent-human somatic cell hybrids, each of which contained a single human chromosome [29]. In *A. thaliana*, however, rDNA clusters can be effectively unlinked from each other in experimental populations.

The genome of *A. thaliana* contains two rDNA clusters located at the top of chromosomes 2 and 4, hereinafter referred to as rDNA-2 and rDNA-4, respectively (Fig. 1a) [30, 31]. In the reference accession Col-0, only rDNA-4-derived rRNA genes are actively transcribed, while rDNA-2 is silent [32]. However, 45S rRNA gene copy number varies massively among natural accessions of *A. thaliana* [33–35], with both rDNA clusters contributing [36]. Furthermore, considerable variation in the degree of DNA methylation at rDNA clusters has also been observed, suggesting variation in silencing [37–39]. Here we exploit sequence variation among natural lines [40] to investigate whether there is also variation in rDNA cluster expression among *A. thaliana* accessions.

## Results

### Sequence variation in rRNA genes within and between individuals

Monitoring expression of particular rRNA genes is difficult because all copies are extremely similar due to concerted evolution, an evolutionary process that promotes homogeneity among the many rRNA gene repeats [41–45]. Nonetheless, by taking advantage of next-generation DNA sequencing data from the 1001 Genomes Consortium [40], we identified 2264 polymorphic sites along a 7.7 kb transcribed portion of the 45S rRNA gene, spanning from the minimal promoter to the end of the 25S rRNA subunit (see “Methods”; Fig. 1b and Additional file 1). Sites can have multiple variants; thus, there are 2844 variants found in at least one individual (the alternative variant must be present in at least 5% of the individual’s total 45S rRNA genes). Note that these are rRNA gene variants within as well as between individuals: while 56% of the variants are specific to one accession, 249 (9%) of them are shared by at least ten accessions (Fig. 1b). Interestingly, sequence variation is not restricted to the external transcribed spacer (ETS) or the internal transcribed spacer (ITS) sequences as previously reported for Col-0 [32, 46], but is also present within the highly conserved ribosomal subunits (although the population frequency of such variants is clearly lower, suggesting the action of purifying selection; see Fig. 1b).

Our primary interest in these polymorphisms is to use them as markers to monitor rDNA cluster-specific expression. In order to assign them to rDNA clusters, we used the multi-parent advanced generation inter-cross (MAGIC) population: a set of recombinant inbred lines derived from intercrossing a genetically heterogeneous stock of 19 worldwide accessions [47]. In the MAGIC population, the two rDNA clusters have been effectively randomized with respect to each other. In addition, due to the lack of recombination between homologous rDNA clusters in *A. thaliana* [30, 31, 48], rDNA clusters and flanking regions are usually inherited as haplotype blocks, making it possible to infer rDNA cluster identity from single-nucleotide polymorphism (SNP) markers in the flanking regions [36]. To ensure that all rRNA gene variants could be mapped, we used only variants unique to or shared by less than eight of the 19 founder accessions [49]. Through standard linkage mapping and manual curation (see “Methods”) in this population, we identified rDNA cluster-specific markers in the founder accessions (Fig. 1c and d, Additional file 2: Table S1).

### Accessions express either rDNA-2 or rDNA-4, or both

**Fig. 2:**
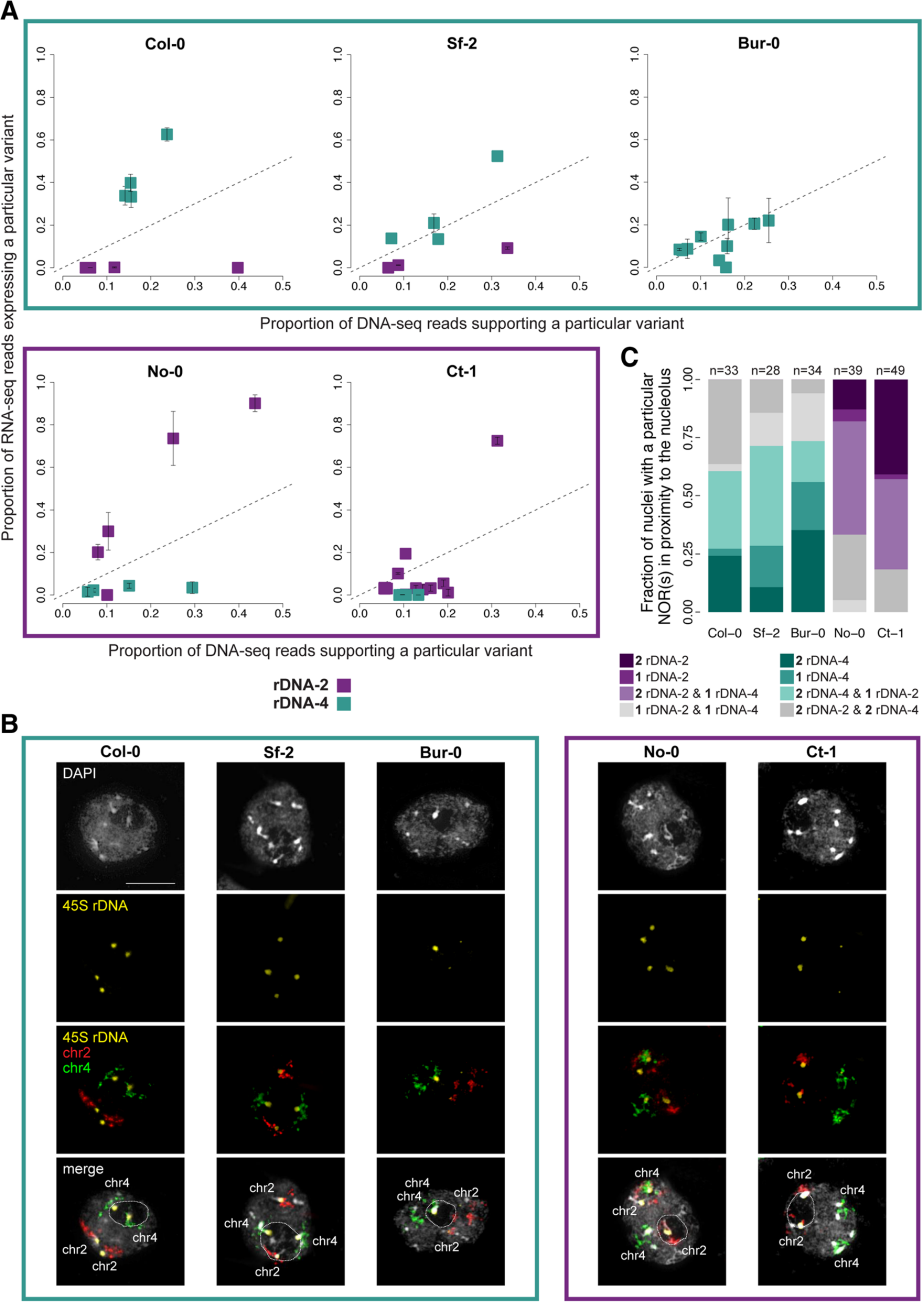
rDNA cluster-specific expression in natural inbred lines. **a** The proportion of RNA-seq reads expressing a particular reporter variant (*y-axis*) against the proportion of DNA-seq reads accounting for the existence of the same variant (*x-axis*) for five natural inbred lines: Col-0 (6909), Sf-2 (7328), Bur-0 (7058), No-0 (7273), and Ct-1 (7067). Notice that no variants Bur-0 rDNA-2-specific passed the threshold (present in > 5% of copies within an individual) due to the small size of that rDNA cluster (Fig. 2b and [36]). *Error bars* represent standard deviations of three to seven biological replicates. The *dashed line* indicates the one-to-one ratio between DNA and RNA. **b** FISH results for the same accessions as in (**a**) showing that rDNA clusters carrying actively transcribed rRNA localize in proximity to the nucleolus, while silenced rDNAs are observed elsewhere in the nucleus. Images in *black* and *white* show DAPI-stained nuclei. Probes hybridizing the 45S rRNA gene cluster, chromosomes 2 and 4 are highlighted in *yellow*, *red*, and *green*, respectively. The nucleolus is marked by a dashed contour. Bar = 10 μm. For (**a**) and (**b**), *purple* and *turquoise* colored frames indicate accessions dominant for rDNA-2 and rDNA-4, respectively. **c** Relative frequency of nuclei with a particular rDNA configuration in relation to the nucleolus for the same parental accessions as in (**a**) and (**b**). The colored areas correspond to nuclei displaying exclusively two rDNA-2 (*dark purple*), one rDNA-2 (*mid purple*), two rDNA-2 per one rDNA-4 (*light purple*), two rDNA-4 (*dark turquoise*), one rDNA-4 (*mid turquoise*), two rDNA-4 per one rDNA-2 (*light turquoise*), one rDNA-2 per rDNA-4 (*light gray*), and two rDNA-2 per two rDNA-4 (*light green*) hybridization signals localized to the nucleolus. The number of nuclei (n) analyzed per accession is indicated at the top of each bar

We monitored the expression of these rDNA cluster-specific variants in leaves of approximately two-week old seedlings from different accessions. In agreement with previous results [32], none of the four rDNA-2-specific variants were expressed in the reference accession Col-0 (6909), while all rDNA-4-specific variants were (Fig. 2a and Additional file 3). Since active rRNA genes in *A. thaliana* are present in nucleoli when active and excluded when silenced [11], we reasoned that rDNA clusters carrying active variants would localize in proximity to the nucleolus more frequently than silenced ones (Fig. 2b). Indeed, rDNA cluster localization by means of fluorescence in situ hybridization (FISH) revealed that Col-0 rDNA-4 preferentially associated with the nucleolus in 61% of cells, while in the other nuclei (39%) rDNA-4 and rDNA-2 were equally close to the nucleolus (Fig. 2c and Additional file 4).

**Fig. 3:**
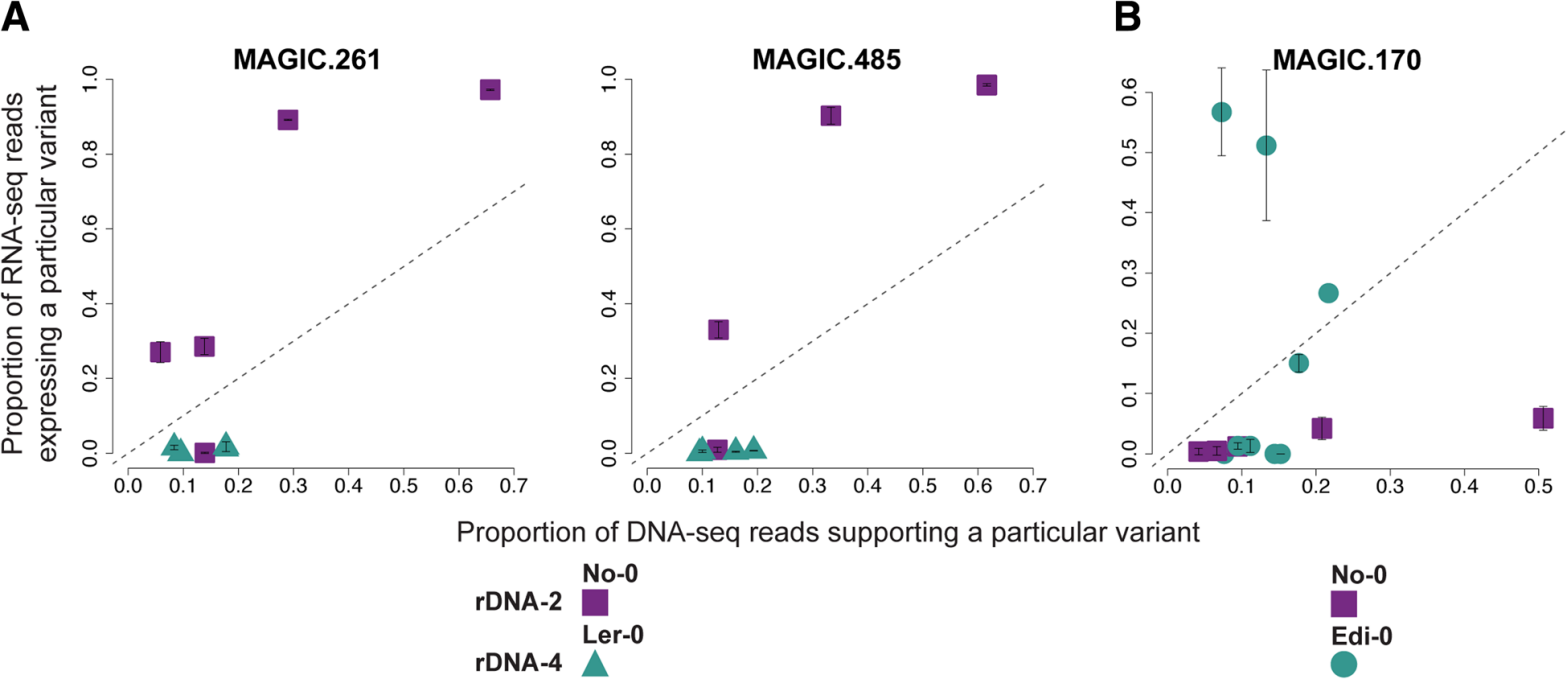
The genotype at both rDNA clusters determines rRNA gene expression. **a** The proportion of RNA-seq reads expressing a particular reporter variant (*y-axis*) against the proportion of DNA-seq reads accounting for the existence of the same variant (*x-axis*) for MAGIC lines 261 and 485. **b** Similar to (**a**), but for MAGIC line 170. For both subfigures, *error bars* represent standard deviations of two biological replicates and the *dashed line* indicates the one-to-one ratio between DNA and RNA

However, what is true for Col-0 is not universal (Fig. 2). While five other accessions—Sf-2, Bur-0, Edi-0, Ws-0, and Wu-0—appear to behave like Col-0 in that rDNA-4 is expressed and rDNA-2 silenced, four accessions—No-0, Ct-1, Can-0, and Hi-0—show the opposite pattern, with rDNA-2 exclusively expressed, and two lines—Ler-0 and Zu-0—express both rDNA-2 and rDNA-4 (Fig. 2 and Additional file 5: Figure S1). Furthermore, analysis of the MAGIC lines allowed us to determine the expression of the parental rDNA clusters in different combinations. Remarkably, the results strongly suggest that the expression at one rDNA cluster depends on the genotype at both clusters (Fig. 3). For example, in accession No-0 only rDNA-2 is expressed while in Ler-0 both rDNA clusters are (Fig. 2 and Additional file 5: Figure S1); however, in MAGIC lines 261 and 485, rDNA-4 inherited from Ler-0 is silenced when combined with rDNA-2 from No-0 (Fig. 3a). Thus, No-0 rDNA-2 expression is dominant over that of Ler-0 rDNA-4. In contrast, when No-0 rDNA-2 and Edi-0 rDNA-4 are combined in MAGIC line 170, the former is silenced and the latter expressed (Fig. 3b), despite the fact that both are expressed in the parental lines (Fig. 2 and Additional file 5: Figure S1). Thus, Edi-0 rDNA-4 expression is dominant over No-0 rDNA-2. Moreover, based on our limited data, the pattern of silencing appears to be deterministic (rather than stochastic) as suggested by MAGIC lines that after undergoing independent pedigrees—five generations of intermating [50]—inherited the same genotypes at both rDNA loci and display similar expression status (Fig. 3a, Additional file 5: Figure S2).

These results lead to two conclusions. First, the expression pattern in Col-0—rDNA-4 expressed and rDNA-2 silenced—is clearly not a general feature. Indeed, in Cvi-0 X Ler-0 recombinant inbred lines (RILs) expression of both rDNA clusters was observed in lines in which Cvi-0 contributed rDNA-2 and Ler-0 rDNA-4 [51], suggesting that rDNA-4 is not always fully dominant over rDNA-2. Equally importantly, the varying pattern of expression suggests that dominance is not a property of the rDNA cluster per se, but rather of its “allelic” content (i.e. haplotypic—note that rDNA clusters and flanking regions are usually inherited as complete haplotypes since homologous recombination is suppressed [48]). Dominance should thus occur between different haplotypes in individuals heterozygous with respect to a particular rDNA cluster.

### Dominance is a property of rDNA “alleles”

**Fig. 4:**
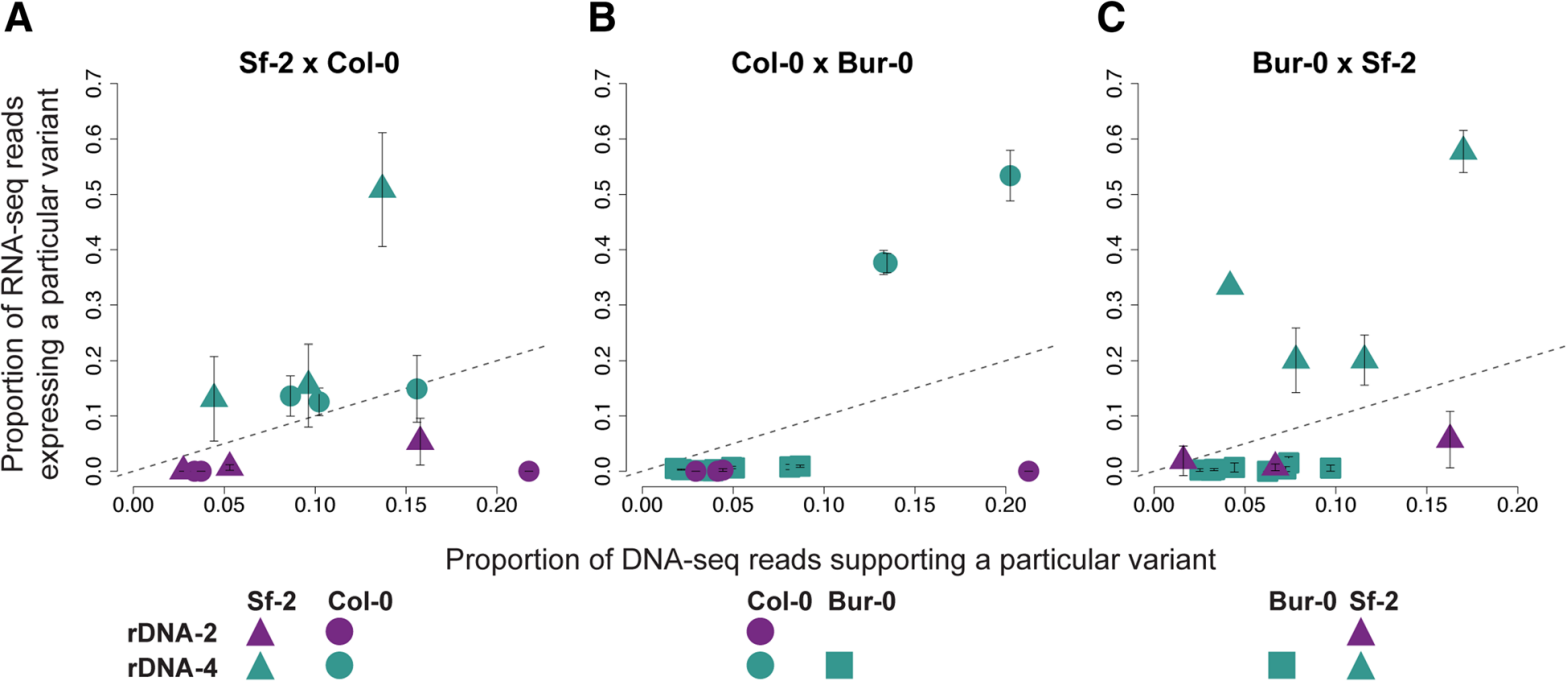
“Every rDNA cluster for itself” in F_1_ crosses. **a** The mean proportion of RNA-seq reads expressing a particular reporter variant (*y-axis*) against the proportion of DNA-seq reads accounting for the existence of the same variant (*x-axis*) for F_1_: ♀ Sf-2 x ♂ Col-0. **b** Similar to (**a**), but for F_1_: ♀ Col-0 x ♂ Bur-0. **c** Similar to (**a**) and (**b**), but for F_1_: ♀ Bur-0 x ♂ Sf-2. The absence of reporter variants Bur-0 rDNA-2-specific is explained in the legend of Fig. 2b. For all subfigures, *error bars* represent standard deviations of four biological replicates. The *dashed line* indicates the one-to-one ratio between DNA and RNA

Since the MAGIC lines are inbred, they can only be used to study interactions between loci (i.e. epistasis). To investigate interactions between alleles on homologous chromosomes (i.e. classical dominance), we crossed accessions with dominant rDNA-4 and analyzed the hybrid F_1_ plants. In these crosses, Sf-2 rDNA-4 and Col-0 rDNA-4 seem to be co-dominant (Fig. 4a), while Sf-2 and Col-0 rDNA-4 are both dominant over Bur-0 rDNA-4 (Fig. 4b and c)— we detected weak expression of Bur-0 rDNA-4 in some replicates when Bur-0 is used as a mother and Col-0 as a father (see Additional file 5: Figure S3). Thus, dominance can occur not only between rDNA clusters on different chromosomes, but also between rDNA “alleles” of the same rDNA cluster.

### Genetic analysis of interactions between rDNA “alleles”

**Fig. 5:**
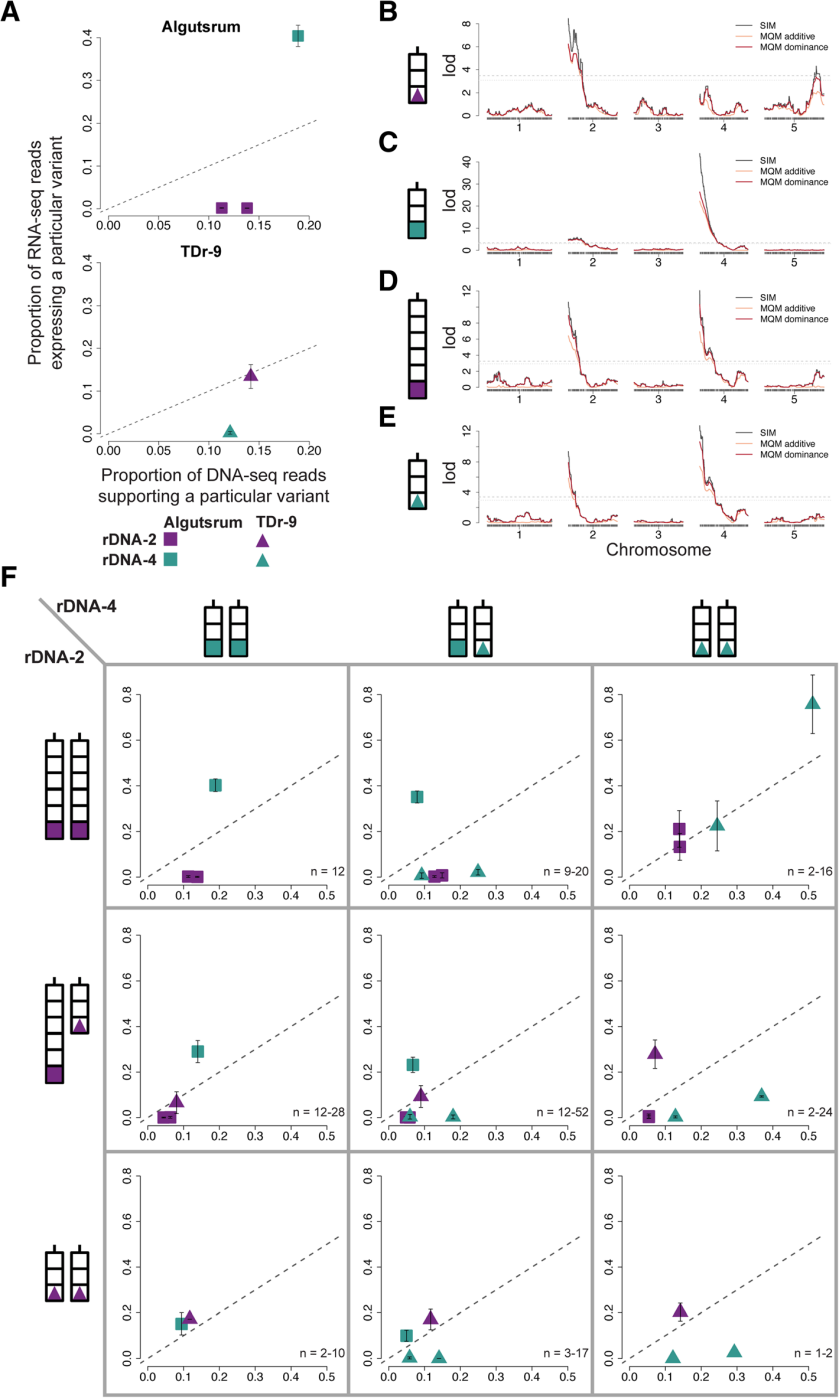
Genetic evidence of the interaction between rDNA clusters. **a** The proportion of RNA-seq reads expressing a particular reporter variant (*y-axis*) against the proportion of DNA-seq reads accounting for the existence of the same variant (*x-axis*) for parental accessions Algutsrum (8230) and TDr-9 (6195). *Error bars* represent standard deviations of two biological replicates. The *dashed line* represents the one-to-one ratio between DNA and RNA. **b** Linkage mapping of the expression of a TDr-9 rDNA-2-specific variant (position 1861 in the ETS, T to G) in 68 F_2_ individuals derived from the selfed F_1_ progeny of a cross between ♀ Algutsrum x ♂ TDr-9. **c** Similar to (**b**), but for a Algutsrum rDNA-4-specific variant (position 2445 in the 18S, T to C) in 183 F_2_ individuals. **d** Similar to (**b**) and (**c**), but for a Algutsrum rDNA-2-specific variant (position 3904 in the 18S, C to G) in 176 F_2_ individuals. **e** Similar to (**b**–**d**), but for a TDr-9 rDNA-4-specific variant (position 4078 in the ITS, C to deletion) in 162 F_2_ individuals. For subfigures (**b**–**e**), *black lines* indicate Simple Interval Mapping (SIM), while *orange* and *red lines* indicate Multiple-QTL Mapping (MQM) additive and dominance models, respectively. The *horizontal solid* and *dashed gray lines* correspond to the permutation test at 10% and 5% significance levels, respectively. **f** Schematic representation of the rDNA cluster combinations inherited by F_2_ individuals (cross-matches between schemes outside the matrix), and the resulting mean expression pattern of their rRNA genes according to all available rDNA cluster-specific variants (squares inside the matrix). *Error bars* represent standard deviations across the number of F_2_ individuals (n, which varies between different reporter variants and is given as a range in the figures) in each rDNA cluster combination. *Dashed lines* indicate the one-to-one ratio between DNA and RNA

To gain further insight into the complex interactions among rDNA “alleles,” we carried out linkage mapping in an F_2_ population derived from a cross between Algutsrum (8230), in which rDNA-4 is dominant, and TDr-9 (6195), in which rDNA-2 is dominant (Fig. 5a, Additional file 2: Table S2 and Additional file 6). Expression of each of the four rRNA gene clusters was mapped separately (Fig. 5b–e). Mapping identified the distal end regions of chromosomes 2 and 4, i.e. the location of the rDNA clusters, as the major source of variation for the expression of TDr-9 rDNA-2 and Algutsrum rDNA-4, respectively (Fig. 5b and c). These rDNA clusters, both dominant in their parental lines, are expressed as long as they are inherited: even when both rDNA clusters are present, their rRNAs co-exist (they are co-dominant) (Fig. 5f). However, expression of the rDNA clusters that were silenced in the parental lines, i.e. Algutsrum rDNA-2 and TDr-9 rDNA-4, is clearly influenced by the full two-locus genotype (Fig. 5d and e). Both rDNA clusters are recessive relative to their “allelic” rDNA counterparts: they are only expressed in the presence of each other in homozygous state (Fig. 5f).

In fact, these results are consistent with the study of Riddle and Richards [38], who detected an epistatic interaction between rDNA loci when mapping overall levels of DNA methylation at rDNAs in a cross between Can-0 and Col-0. However, in an F_2_ population derived from a cross between Cvi-0 and Col-0 rDNA methylation levels segregated as a Mendelian additive trait. Since DNA methylation and repressive chromatin modifications are needed for rRNA gene silencing [14, 52–55], it is plausible that this study indirectly mapped overall rRNA expression, and that an interaction was detected in the former population because Can-0 is dominant for rDNA-2 (Additional file 5: Figure S1) and Col-0 is dominant for rDNA-4 (Fig. 1), while in the second population no interaction was detected because both parents (Col-0 and Cvi-0) are dominant for rDNA-4 (Additional file 2: Table S3, Additional file 5: Figure S4 and Additional file 7).

## Discussion

We are discovering huge amounts of variation in 45S rRNA genes on every level. At the gross level of total copy number, variation in 45S rRNA gene copy number is largely responsible for an over 10% variation in genome size among *A. thaliana* accessions [35] and the relative size of the two rDNA clusters varies greatly among accessions [36]. At the sequence level, there is variation in the conserved catalytic subunits themselves both within and among accessions. Furthermore, these rRNA gene variants readily express and make for a heterogenous rRNA pool in the cell, the functional significance of which is completely unknown. Ribosome heterogeneity has been studied mainly in the context of the regulation of the ribosomal proteins, the diversity and activity of other ribosome-associated factors and, although not fully understood, the modifications that the rRNA subunits suffer after transcription [56, 57]. In eukaryotes, there have been few attempts to study heterogeneity at the sequence level of the rRNA subunits: in the parasite *Plasmodium* two structurally distinct 18S rRNAs are differentially expressed during its life cycle [58, 59]; in humans, the 28S rRNAs have been shown to be heterogeneous in both mono- and polysomal fractions [26, 60]; similarly, in both the sea urchin *Paracentrotus lividus* [61] and *A. thaliana* several transcribed 5S rRNA variants are readily incorporated in functional ribosomes [62]. Our study provides the most comprehensive catalogue of rRNA gene variants to date, and will hopefully be useful for investigating their possible adaptive role, either at the level of transcriptional regulation, rRNA stability or translational efficiency.

Irrespective of their functional significance, these variants, although rarely homogenized throughout an entire rDNA cluster, can be used as markers of the expression of a particular rDNA cluster. Our findings make it clear that the silencing phenomenon known as nucleolar dominance occurs both within [32] and among natural lines of *A. thaliana* [51]. Furthermore, we demonstrate that dominance is a property neither of the parental strain nor of the chromosomal position (i.e. chromosome 2 versus 4), but rather of the specific “allelic” content of each rRNA gene cluster—including, perhaps, flanking DNA. Indeed, a recent study implicates centromere-proximal sequences in this regulation [63].

However, the molecular basis of the epistatic and allelic interactions among rDNA clusters remains unknown. Interestingly, rDNAs derived from different species in the genus *Brassica* follow a hierarchical dominance relationship [13] similar to the one among the many alleles at the self-incompatibility locus (*S*-locus) in Brassicaceae [64]. In *Arabidopsis halleri,* dominance at the *S*-locus is largely controlled by a set of small non-coding RNAs produced by dominant *S*-alleles that target a repertoire of more recessive *S*-alleles resulting in their epigenetic silencing [65, 66]. Since uniparental rRNA gene silencing involves short interfering RNAs (siRNAs)-directed DNA methylation (RdDM) pathway proteins in the hybrid plant *Arabidopsis suecica* [67, 68] and there is evidence that non-coding RNAs can act *in trans* to silence other rRNAs gene repeats in mice [69–71], it is tempting to speculate that a similar mechanism to the one described for the *S*-locus might explain how the rDNA clusters “talk” to each other. Surprisingly, in *A. thaliana* Col-0 RdDM pathway mutants RNA polymerase IV (*nrpd1*), Dicer-like-3 (*dcl2/3/4*), and DNA methyltransferases DRM1 and 2 (*drm1/2*) have, if any, a negligible effect on disrupting silencing of rDNA-2 (Additional file 5: Figure S5; [11]). In contrast, DNA maintenance methyltransferase MET1 (the ortholog of mammalian DNMT1), which is responsible for cytosine methylation in the CG context independently of siRNAs, is needed to silence rDNA-2 [11]. However, these results do not necessarily exclude the involvement of the RdDM pathway in the silencing of rDNA clusters in *A. thaliana*. In *A. suecica*, for instance, re-establishment of nucleolar dominance was observed in T2 progeny of one of the three RNA-dependent RNA polymerase *RDR2*-RNAi lines [67]. As appears to be the case for transposable elements, the establishment of silencing may well be distinct from its maintenance and many semi-redundant mechanisms may be involved [72].

## Conclusions

We show here that rRNA gene expression in *A. thaliana* varies greatly between accessions, with some expressing one rDNA cluster, some the other, and some both. We further show that this expression is regulated via complex epistatic and allelic interactions between rDNA cluster haplotypes that apparently control the entire array via an unknown mechanism. Our paper represents a major new finding in the regulation of rRNA genes and points to several exciting questions about the underlying molecular mechanisms and evolutionary rationale for their existence, especially in context of our recent discovery of massive copy-number variation between accessions.

## Methods

### Plant material and growth conditions

For expression analyses corresponding to the founders of the MAGIC population [49], MAGIC lines [73], and accession Cvi-0 [74, 75], we obtained publicly available messenger RNA (mRNA)-seq data (an overview of the library types and read lengths are provided in Additional file 8). Similarly, for identification of rRNA gene variants at the DNA level in natural accessions [40, 49, 76], the MAGIC population [73], and the Cvi-0 X Ler-0 RIL population [36], we used data released elsewhere (Additional file 8).

For the F_1_ crosses (♀ Col-0 x ♂ Bur-0, ♀ Bur-0 x ♂ Col-0, ♀ Col-0 x ♂ Sf-2, ♀ Sf-2 x ♂ Col-0, and ♀ Bur-0 x ♂ Sf-2), we harvested leaves from one two-week-old and three three-week-old plants per cross for total RNA-sequencing (RNA-seq). Additionally, we DNA-sequenced two individuals per cross.

The F_2_ population was derived from the selfed F_1_ progeny of a cross between accessions ♀ Algutsrum (8230) x ♂ TDr-9 (6195). F_2_ seeds were stratified for five days in 0.1% agarose at 4 °C. For RNA isolation, aerial portions of the plants were harvested at the nine true leaf stage 7.5–8.5 h into the light period. We sequenced individuals of the F_2_ population with two different protocols: one designed for mRNA-seq (183 individuals) and another one for total RNA-seq (a subset of 162 individuals, see below). For DNA-sequencing, we harvested leaves from other 16 F_2_ individuals.

Mutant lines *dcl2/3/4* (alleles *dcl2-1*, *dcl3-1*, and *dcl4-2*) and *nrpd1a-3* used in this study have been described previously [77–80]. We harvested leaves from three-week-old plants in three biological replicates. All plants were grown under long photoperiod conditions (16 h light and 8 h dark) at 16 °C.

### RNA isolation and library preparation

For the F_1_ crosses and the RdDM mutants, RNA was isolated with TRIzol® Reagent (#15596-018, Ambion®) according to manufacturer’s instructions. Contaminating genomic DNA was removed with the Turbo DNA-*free*™ kit (#AM1907, Ambion®), then TURBO DNase was inactivated with DNase Inactivation Reagent and centrifuged at 10,000 × g for 2 min. RNA concentration was quantified using a NanoDrop 1000 spectrophotometer (Thermo Scientific).

For the F_2_ population, RNA was isolated using the methods described in Gan et al. (supplementary methods, 7.2) [49]. Briefly, individual frozen plants were ground in liquid nitrogen using a mortar and pestle and RNA was isolated using PureLink Plant RNA Reagent (#12322-012, Invitrogen, Carlsbad, CA, USA) following the manufacturer’s instructions. Precipitated RNA was resuspended in RNAsecure Resuspension Solution (#AM7010, Ambion®; Life Technologies, Carlsbad, CA, USA) following manufacturer’s guidelines to prevent RNA degradation. Contaminating genomic DNA was removed by incubating samples with Turbo DNase (#AM2238, Ambion®) for 15 min at 37 °C. Finally, RNA was precipitated using 2.5 volume of 100% ethanol, 0.1 volume 5 M Ammonium Acetate, and 1 μL glycogen (5 mg/mL) and resuspended in RNase-free water. RNA concentration was quantified using a NanoDrop 2000 spectrophotometer (Thermo Scientific, Waltham, MA, USA) and RNA integrity was determined using an RNA 6000 nano assay (Bioanalyzer 2100, Agilent Technologies, Palo Alto, CA, USA).

#### F_2_ mRNA-seq library preparation and sequencing

RNA-seq libraries were constructed using the Illumina TruSeq RNA Sample Preparation Kit V2 (# 15026495 Rev. C). Two kits of each barcode set were used for a total of 183 libraries. Libraries were prepared following the manufacturer’s instructions. Briefly, poly-A mRNA was purified from 1 μg of isolated total RNA using poly-T oligo-attached magnetic beads. Purified RNA was fragmented into approximately 120–210 bp fragments (average size ~155 bp) using divalent cations at 94 °C for 8 min. After fragmentation, first strand complementary DNA (cDNA) synthesis was performed using random primers and reverse transcriptase, followed by second strand cDNA synthesis using DNA polymerase I and RNase H. The cDNA fragments then went through end-repair and overhang A addition reactions followed by the ligation of the RNA Adapters. Finally, the products were purified and enriched using 12 polymerase chain reaction (PCR) cycles.

Libraries were validated using the DNA 1000 assay (Bioanalyzer 2100, Agilent Technologies, Palo Alto, CA, USA) and library concentrations were determined for all cDNA fragments between 200 and 700 bp. Libraries were normalized to 10 nM. Sets of 24 barcoded libraries were pooled into a single lane of an Illumina HiSeq2000 instrument and run as 50 bp single-end (SE) reads. Samples were run at the Microarray Core Facility at the Huntsman Cancer Institute (University of Utah, USA).

#### F_1_ and F_2_ total RNA-seq library preparation and sequencing

We prepared libraries using the SENSE Total RNA-Seq Library Prep Kit (Lexogen) according to the manufacturer’s instructions with the following specifications: 200 ng of total RNA were used as input; the sample was incubated at 37 °C for 110 min. In the reverse transcription and ligation step, after the second strand synthesis step, 27 μL of Purification Solution were added in the first purification step to increase the fraction of inserts < 200 nt, and 16 cycles were used during the amplification step.

Libraries were validated with a Fragment Analyzer™ Automated CE System (Advanced Analytical), concentrations were determined for all fragments between 200 and 700 bp, and pooled in two sets at equimolar concentration for 96-multiplex sequencing. Libraries were sequenced on an Illumina HiSeq™ 2500 Analyzer using manufacturer’s standard cluster generation and sequencing protocols in 100 bp SE mode. Samples were run at the at the Vienna Biocenter Core Facilities (VBCF) NGS unit in Vienna, Austria (http://www.vbcf.ac.at).

Removal of adapter contamination was performed with BBDuk from the BBMap package (v35.10; B. Bushnell, http://sourceforge.net/projects/bbmap/). The first 11 and the last six nucleotides of every read were further removed with Seqtk (H. Li, https://github.com/lh3/seqtk). Finally, reads shorter than 50 bp were removed with Trimmomatic (v0.33) [81].

### DNA isolation and library preparation

We extracted DNA with the NucleoMag® 96 Plant (Macherey-Nagel) kit according to the manufacturer’s instructions. We prepared libraries using the Illumina Nextera™ Kit. Standard Nextera library construction was modified to reduce volume in the protocol. Briefly, tagmentation reaction was set up to 2.5 μL final volume with 2.5 ng of input DNA. For PCR amplification and multiplexing, we used Nextera Index Kit (Illumina) indexes. Size selection and PCR clean-up were performed with Agencourt AMPure XP Beads (Beckman Coulter). After PCR enrichment, libraries were validated with Fragment Analyzer™ Automated CE System (Advanced Analytical) and pooled in equimolar concentration for 96X-multiplex. Libraries were sequenced on Illumina HiSeq™ 2500 using manufacturer’s standard cluster generation and sequencing protocols in 125 bp paired-end (PE) mode at the VBCF in Vienna, Austria.

### Identification of 45S rRNA gene variants at the DNA level

#### 1138 natural accessions

In addition to the 1135 accessions sequenced by the 1001 Genomes Consortium [40], we analyzed accessions Mt-0 (6939), Po-0 (7308), and Hi-0 (8304), which are among the 19 founders of the MAGIC population [49]. We also substituted the reference accession Col-0 (6909) from the 1001 Genomes Consortium for another Col-0 of better sequencing quality [76].

For each genome, we performed 3’ adapter removal (either TruSeq or Nextera), quality trimming (quality 15 and 10 for 5’ and 3’-ends, respectively) and N-end trimming with cutadapt (v1.9) [82]. Then, we mapped all PE reads separately to a single 45S rRNA gene reference (described in [36]) and to the *A. thaliana* TAIR10 reference genome with BWA-MEM (v0.7.8) [83, 84]. We used Samtools (v0.1.18) to convert file formats [85] and Sambamba (v0.6.3) to sort and index bam files [86], we removed duplicated reads with Markduplicates from Picard (v1.101) (http://broadinstitute.github.io/picard/), and we performed local realignment around indels with GATK/RealignerTargetCreator and GATK/IndelRealigner functions from GATK (v3.5) [87, 88]. Due to the heterogeneity of Illumina platforms used to sequence these genomes we conducted a base quality recalibration step. We produced base recalibration reports based on the alignment to TAIR10 reference by providing known indels and SNPs from the 1001 Genomes Consortium to the function GATK/BaseRecalibrator; recalibrated base qualities were updated in the reads aligned to the 45S rDNA reference with function GATK/PrintReads in BQSR mode.

To detect polymorphisms along the 45S rDNA we retrieved per-site information with both the function variation_strand from the python package pysamstats (v0.24.2; A. Miles, https://github.com/alimanfoo/pysamstats) and a patched version of it that filters out bases with base quality lower than 20. While we used the output of the former to count the proportion of alternative alleles at each reference position, we used the output of the latter to calculate the strand bias (SB) score at each variable site according to the formula: $\left|\;\frac{b}{a+ b}-\frac{d}{c+ d}\right|/\left(\frac{b+ d}{a+ b+ c+ d}\right)$, where $a$ and $c$ represent the forward and reverse strands’ counts for the major allele, respectively, and $b$ and $d$ represent the forward and reverse strands’ counts for the minor allele, respectively [89]. For each accession, we excluded from further analyses (i.e. mapping in the MAGIC population and expression analysis) variable sites with a SB score higher than 0.8. Similarly, we excluded alternative variants supported by less than 5% of the reads spanning that position within a given genome. We only report variants lying along a 7.7 kb transcribed portion of the 45S rDNA, spanning from the minimal promoter to the end of the 25S rRNA subunit in our 45S reference gene (300–8009 bp) [36]. Finally, at sites for which an alternative variant is more frequent than the reference allele, we also analyzed the reference allele (Additional file 1).

#### MAGIC lines, F_1_ individuals, F_2_ population, and RILs

We mapped low-coverage DNA-seq PE-reads of 393 MAGIC individuals, 10 F_1_s, 16 F_2_s, and eight RILs (see below) as described above for data from the 1001 Genomes Consortium, with the exception of the base recalibration step. Similarly, to detect polymorphisms we employed the same pipeline described above and only report variants that have been validated in the original parental accessions.

### Annotation of rDNA cluster-specific variants

#### Founders of the MAGIC population

We focused on variants shared by seven or fewer of the 19 founder accessions and used two complementary approaches. First, we simply performed linkage mapping and multiple imputation of each variant in the 393 individuals of the MAGIC population with R/happy [47, 90]. Second, we used the software “reconstruction” [73] to infer the genotypes at the top of chromosomes 2 and 4, and compared this result with the information recovered from the rRNA gene variants themselves in each MAGIC line (see above; Additional file 3). For variants shared by few accessions the results of the first analysis alone were sufficient to determine rDNA cluster-specificity (Fig. 1c and d); however, for variants shared among many accessions both analyses were required to produce unambiguous results and correct wrongly inferred genotypes. Accession Mt-0 (6939) presented several discrepancies between variants found in the founder line and those found in MAGIC lines, thus was excluded from the study. rDNA cluster-specific markers for the founder accessions are reported in Additional file 2: Table S1.

#### Parents of the F_2_ population

We selected variants supported by at least 10% of the reads spanning that position in a parental line and absent in the other. We performed genotyping by low-coverage DNA-sequencing as described in Rabanal et al. [36] binning SNP markers in 100 kb windows. rDNA cluster-specificity was unambiguously inferred from the predicted genotypes at the top of chromosomes 2 and 4 (Additional file 6). rDNA cluster-specific markers for the parental accessions are reported in Additional file 2: Table S2.

#### Accession Cvi-0

We selected variants present in Cvi-0 and absent in Ler-0. rDNA cluster-specificity was unambiguously inferred from the predicted genotypes at the top of chromosomes 2 and 4 in 16 re-sequenced RILs (CVL5, CVL9, CVL10, CVL13, CVL17, CVL20, CVL34, and CVL38) derived from a cross between Cvi-0 and Ler-0 [36, 91] (Additional file 7). rDNA cluster-specific markers for accession Cvi-0 are reported in Additional file 2: Table S3.

### Expression of 45S rRNA gene variants

To avoid any bias derived from aligning or calling variants, we mapped reads (whether single-end or PE) as described for low-coverage DNA-sequencing reads, with the exception of the removal of duplicated reads step. To detect polymorphisms, due to the heterogeneous nature of the multiple datasets (strand-specific and non-strand-specific), no filter for SB was applied. However, we only report variants at the RNA level that have been validated as variants at the DNA level. In addition, we applied a minimum cutoff of 25 reads covering a given variable site to report it at the RNA level.

Despite selection with poly-T oligo-attached beads during library preparation, the mRNA-seq datasets used in this study still contain at least 6.1% of reads mapping to the 45S rRNA gene, on average (see Additional file 8). This should be contrasted with libraries from total RNA, which contain more than 60% of reads mapping to the 45S rRNA gene, on average. Low expressed ETS and ITS regions in particular are differentially covered by each type of library. Nonetheless, results based on well-covered variants are consistent between the different types of libraries.

### Statistical genetics in the F_2_ population

#### Genotyping by RNA-seq

Reads from the individual F_2_ samples were aligned to the TAIR10 reference genome and annotation using the Bowtie (v2.1.0.0)/TopHat (v2.0.8b) [92, 93] pipeline with the following command line parameters: ‘-a 5 -i 5 -I 32000 --b2-very-sensitive --segment-mismatches 2 -g 1’. Employing the Pysam package (v0.7.5; A. Heger, https://github.com/pysam-developers/pysam), we assessed genotypes in each of the 183 F_2_ samples at 266,663 SNP sites polymorphic between the two parents in genes in the TAIR10 annotation, i.e. genotypic data were assessed as the base identities in RNA-seq reads crossing a polymorphic position; the input genotypic data for TDr-9 and Algutsrum were obtained from a previous study [35]. For assessing genotypes at SNP positions near splice sites (e.g. a read aligned across two exons, for which alignment uncertainties are higher), aligned RNA-seq segments exceeding 3 bp were enforced to make a genotypic call; further, SNPs that were called as homozygous for one parent at a rate exceeding tenfold that of the other parent across all 183 samples were also removed from downstream analysis (these may reflect the impact of polymorphisms on read alignments, or alternatively allele-specific expression resulting from structural or other genetic variation, a confounding factor for genotyping with RNA-seq as opposed to DNA-seq reads). Non-overlapping windows of 50 SNPs were used to classify the genomes of each F_2_ individual into heterozygous and homozygous regions. Briefly, for an individual SNP position to be defined as homozygous, over 90% of the RNA-seq reads crossing it had to come from one parent and for the non-overlapping windows of 50 successive SNPs to be classified as homozygous, over 85% of the SNPs in the window had to be assigned to the same parent (else, a window was classified as heterozygous). In F_2_ individuals, only several recombination events are expected per chromosome; thus, long tracks of homozygosity or heterozygosity are expected, and were observed as revealed in plots of the window data across each chromosome for each F_2_ sample. This identified chromosome regions of the same genotype and the breakpoints between homozygous and heterozygous genomic intervals were then manually refined using the genotypic information from single SNPs and/or by inspection of read alignments for given F_2_ samples in IGV [94]. The result of this analysis was that each region of the genome in each F_2_ individual was classified as either homozygous for one or the other parent or alternatively heterozygous. Based on these ranges, the intervening genotypes at all variable positions between the two parents were imputed for use in genetic mapping (535,800 single nucleotide positions in total).

#### Linkage mapping of rDNA cluster expression in the F_2_ population

We performed linkage mapping of the expression of each rDNA cluster-specific variant with the values from the RNA sequencing protocol that covered each variant for the most number of individuals. First, we reduced the original genetic map from 535,800 segregating SNPs to 1394 after dropping those with identical genotype data. Simple interval mapping (SIM) was performed with the R package R/qtl [95]. We further reduced the genetic map to 366 markers with the function pickMarkerSubset with minimum distance of 1. With the latter subset Multiple QTL mapping (MQM) was performed with a 2 centimorgan step size and 20 as window size [96]. One thousand permutations were applied to estimate genome wide significance.

### Fluorescence in situ hybridization (FISH)

We germinated seeds on filter paper soaked in distilled water in a Petri dish at 21 °C. Leaves of two-week-old seedlings grown under long photoperiod conditions (16 h light at 21 °C and 8 h dark at 18 °C) were fixed in ethanol:acetic acid (3:1) fixative at 4 °C for 24 h. The preparation of nuclei spreads from the fixed leaves and FISH followed the protocols published by Mandáková and Lysak [97, 98] with some modifications. Briefly, fixed leaves were rinsed in distilled water and 1 citrate buffer (10 mM sodium citrate, pH 4.8), and digested by 0.3% pectolytic enzymes (cellulase, cytohelicase, and pectolyase) in 1× citrate buffer at 37 °C for 20 min. Digested leaves were placed on a microscopic slide by a Pasteur pipette, disintegrated by a needle in a small drop of 1 citrate buffer, and the material spread in 20 μL of 60% acetic acid on a hot plate (50 °C) for 2 min. After the material was fixed on the slide using 100 μL of the ethanol:acetic acid (3:1) fixative, the slide was tilted and dried using a hair dryer. Prior to FISH, the slide was pretreated by ribonuclease A (100 μg/mL in distilled water) at 37 °C for 1 h and by pepsin (0.1 mg/mL in 10 mM HCl) for at 37 °C for 1 min, and postfixed in 4% formaldehyde in 2× SSC (20× SSC: 3 M NaCl in 0.3 M sodium citrate, pH 7.0) at room temperature for 10 min. The slide was rinsed in 2× SSC between the steps and eventually dehydrated in an ethanol series (70%, 80%, and 96% ethanol, 3 min each). To identify the rDNA clusters, *A. thaliana* BAC clone T15P10 containing 45S rRNA genes was used. To identify *A. thaliana* chromosomes 2 and 4, 11 BAC clones from the upper arm of chromosome 2 (F2I9, T8O11, T23O15, F14H20, F5O4, T8K22, F3C11, F16J10, T3P4, T6P5, and T25N22), and 15 BACs from the upper arm of chromosome 4 (F6N15, F5I10, T18A10, F3D13, T15B16, T10M13, T14P8, T5J8, F4C21, F9H3, T27D20, T19B17, T26N6, T19J18, and T1J1) were used. The 45S rRNA gene probe was labeled with Cy3-dUTP, chromosome 2 BACs with biotin-Dutp, and chromosome 4 BAC clones with digoxigenin-dUTP by nick translation [98]. A total of 100 ng from each labeled BAC DNA were pooled together, ethanol precipitated, dissolved in 20 μL of 50% formamide in 10% dextran sulfate in 2× SSC and pipetted on the selected spreads of nuclei. The nuclei and DNA probe were denatured together at 80 °C for 2 min and the slide incubated at 37 °C overnight. Hybridized probes were visualized either as the direct fluorescence of Cy3-dUTP (yellow) or through fluorescently labeled antibodies against biotin-dUTP (red) and digoxigenin-dUTP (green). After FISH, the slide was counterstained with 4,6-diamidino-2-phenylindole (DAPI, 2 μg/mL) in Vectashield antifade. Fluorescence signals were analyzed and photographed using a Zeiss Axioimager epifluorescence microscope and a CoolCube camera, and pseudocolored using Adobe Photoshop CS5 software. Darker, less DAPI-stained areas within the photographed nuclei, corresponding to the nucleoli, were demarcated in Adobe Photoshop. rDNA clusters (visualized by the 45S rRNA gene probe) located in the immediate proximity to the nucleoli were counted and evaluated (Additional file 4).

### Reverse transcription polymerase chain reaction (RT-PCR)

Semi-quantitative RT-PCR was performed using cDNA generated with SuperScript III Reverse Transcriptase (Invitrogen) using random hexamers according to the manufacturer’s instructions from 400 ng of total RNA. Two microliters of cDNA were used for PCR amplification of rRNA gene 3' variable region (VAR1-4; 30 cycles) and ACT2 (26 cycles). For VAR1-4 [28] we used primers 5'-GAG ACA GAC TTG TCC AAA ACG CCC AC-3' and 5'-CTG GTC GAG GAA TCC TGG ACG ATT-3', while for ACT2 primers 5'-AAG TCA TAA CCA TCG GAG CTG-3' and 5'-ACC AGA TAA GAC AAG ACA CAC-3' [11].

## Additional files

Additional file 1: 45S rRNA gene variants analysis for the 1138 genomes. Variable sites along the 45S rRNA gene present in at least 5% of the copies within an individual for 1138 accessions. Nomenclature: X[*position*].[*reference*].[*alternative*]. (CSV 39979 kb)

Additional file 2: **Table S1.** rDNA cluster-specific markers in the 19 founders of the MAGIC population. **Table S2.** rDNA cluster-specific markers in the parental accessions of the F_2_ population: Algutsrum and TDr-9. **Table S3.** rDNA cluster-specific markers in accession Cvi-0. (PDF 105 kb)

Additional file 3: 45S rRNA gene variants analysis for the MAGIC population and founders. Variable sites along the 45S rRNA genes at the DNA and RNA level for the founders of the MAGIC population, the MAGIC lines, and the F_1_ crosses used in this study. For the MAGIC lines, genotypes at the top of chromosomes 2 (rDNA-2) and 4 (rDNA-4) inferred by the software “reconstruction” [73] and corrected rDNA cluster genotypes according to the variants carried by the rDNA clusters themselves. Type of sequencing-library source is also indicated. Nomenclature: X[*position*].[*reference*].[*alternative*]. (CSV 492 kb)

Additional file 4: Nucleolar association of rDNA-2 and rDNA-4 in five accessions. (PDF 55 kb)

Additional file 5: **Figure S1.** Different accessions express either rDNA-2 or rDNA-4, or both. **Figure S2.** The pattern of rRNA expression is consistent across replicate lines. **Figure S3.** Expression of Bur-0 rDNA-4 when used as a mother in an F_1_ cross. **Figure S4.** Cvi-0 expresses rDNA-4. **Figure S5.** Col-0 rDNA-2 is not reactivated in mutants of the RdDM pathway. (PDF 991 kb)

Additional file 6: 45S rRNA gene variants analysis for the the F_2_ population and parental accessions. Variable sites along the 45S rRNA genes at the DNA and RNA level for the parental accessions of the F_2_ population, and the F_2_ lines used in this study. Predicted genotypes at rDNA-2 and rDNA-4 for each line and type of sequencing-library source are also indicated. Nomenclature: X[*position*].[*reference*].[*alternative*]. (CSV 109 kb)

Additional file 7: 45S rRNA gene variants analysis for accession Cvi-0. Variable sites along the 45S rRNA genes at the DNA and RNA level for the accession Cvi-0. The predicted genotypes at rDNA-2 and rDNA-4 for 8 recombinant inbred lines (RILs; Cvi-0 x Ler-0) and type of sequencing-library source are also indicated. Nomenclature: X[*position*].[*reference*].[*alternative*]. (CSV 4 kb)

Additional file 8: Data availability. (PDF 69.2 kb)
