# Supplementary material for: Epistatic and allelic interactions control expression of ribosomal RNA gene clusters in Arabidopsis thaliana
Source: Genome Biol. 2017 May 3;18:75. doi: 10.1186/s13059-017-1209-z (PMC5414317; doi:10.1186/s13059-017-1209-z)
Supplement: Supplementary file 2 — Table S1. rDNA cluster-specific markers in the 19 founders of the MAGIC population. Table S2. rDNA cluster-specific markers in the parental accessions of the F2 population: Algutsrum and TDr-9. Table S3. rDNA cluster-specific markers in accession Cvi-0. (PDF 105 kb) [file 13059_2017_1209_MOESM2_ESM.pdf]

Additional file 2

Table S1. NOR-specific markers in the 19 founders of the MAGIC population.

Position	Reference	Alternative	NOR2	NOR4	Annotation
343	T	C	Oy-0|Ct-1|Edi-0|Zu-0	Rsch-4|Sf-2|Ct-1|Edi-0|Zu-0	promoter
378	T	C	Tsu-0	Sf-2|Tsu-0	ETS
402	T	C	Wil-2	Wil-2	ETS
404	T	A	Can-0		ETS
405	A	Del	Wil-2	Wil-2	ETS
405	A	C	Can-0		ETS
408	G	A	Ct-1|Hi-0	Zu-0|Hi-0	ETS
413	G	A	Sf-2		ETS
433	G	C	Wu-0		ETS
444	G	A	Col-0|No-0		ETS
449	C	A	No-0|Ws-0		ETS
461	T	C	Can-0		ETS
496	A	C	No-0		ETS
498	A	T	Edi-0		ETS
499	C	G	Wu-0		ETS
507	G	A	Edi-0		ETS
508	C	T	Edi-0		ETS
509	G	C	Ler-0		ETS
516	C	T	Ct-1	Po-0|Ct-1	ETS
526	G	A		Edi-0|Oy-0|Po-0	ETS
557	A	T	No-0|Ws-0		ETS
585	C	G		Edi-0	ETS
593	A	C	Wil-2	Wil-2	ETS
596	G	C		Edi-0|Oy-0	ETS
600	T	C	Wil-2	Wil-2	ETS
608	G	T	Can-0		ETS

610	G	T		Ler-0|Wil-2	ETS
612	G	C		Ws-0	ETS
619	T	C		Edi-0|Oy-0|Po-0	ETS
625	C	T		Ws-0	ETS
639	G	T	Kn-0|Wil-2	Edi-0|Mt-0|Oy-0|Po-0	ETS
650	T	G		Edi-0	ETS
662	G	C		Ler-0	ETS
662	G	T	Wil-2	Wil-2	ETS
670	T	G		Zu-0	ETS
671	T	C		Wu-0	ETS
721	T	C	Ct-1|Hi-0	Bur-0|No-0|Wil-2|Ws-0|Zu-0|Ct-1|Hi-0	ETS
734	G	T	Oy-0		ETS
739	G	T	Col-0		ETS
768	T	C	Can-0	No-0|Ws-0	ETS
770	T	A	Oy-0		ETS
816	T	C	Oy-0|Ct-1|Edi-0|Zu-0	Rsch-4|Sf-2|Ct-1|Edi-0|Zu-0	ETS
877	C	T	Ct-1		ETS
906	C	T		Bur-0	ETS
908	C	Del		Bur-0	ETS
920	A	G	Can-0		ETS
921	T	C	Wil-2	Wil-2	ETS
934	A	C	Kn-0		ETS
965	T	C		Sf-2	ETS
973	T	C	Col-0		ETS
1075	G	A	Kn-0|No-0|Bur-0|Edi-0|Po-0	Hi-0|Oy-0|Sf-2|Bur-0|Edi-0|Po-0	ETS
1078	A	G	Kn-0	Ct-1|No-0	ETS
1105	C	G	Hi-0|Ler-0|Po-0	Edi-0|Wu-0|Ler-0|Po-0	ETS
1146	T	C	Wil-2	Edi-0|Mt-0|Oy-0	ETS
1147	T	C	Hi-0|Zu-0		ETS
1156	T	C		Zu-0	ETS

1157	A	T	Ct-1|Wu-0		ETS
1158	G	C	Kn-0|Wil-2|Mt-0	Mt-0	ETS
1162	A	T	Wil-2	Edi-0|Oy-0	ETS
1166	T	A		Ler-0	ETS
1406	A	T	Can-0		ETS
1417	C	G	Hi-0|Ler-0	Ler-0	ETS
1423	G	Ins	Can-0		ETS
1423	G	T	Can-0		ETS
1452	A	G		No-0	ETS
1459	T	C	Ct-1|Hi-0|Oy-0|Edi-0|Wil-2|Zu-0	Rsch-4|Sf-2|Edi-0|Wil-2|Zu-0	ETS
1466	A	G	Can-0		ETS
1468	T	C		Zu-0	ETS
1494	G	A	Kn-0		ETS
1496	C	Ins	Can-0		ETS
1516	T	C	Oy-0		ETS
1524	T	C		Rsch-4|Sf-2|Tsu-0|Zu-0	ETS
1529	A	T	Can-0|Kn-0	Ler-0|Wil-2|Kn-0	ETS
1596	T	C	Can-0		ETS
1621	T	C	Kn-0	Edi-0|Sf-2	ETS
1683	T	C		Ct-1|No-0|Ws-0	ETS
1698	G	Ins	Kn-0		ETS
1699	T	Ins	Tsu-0	Tsu-0	ETS
1703	T	G	Can-0		ETS
1706	G	A	Hi-0		ETS
1734	T	C	Ct-1		ETS
1742	T	C	Ler-0	Ler-0	ETS
1769	T	C	Oy-0	Col-0|Po-0	ETS
1818	C	T		No-0	ETS
1857	G	A		Edi-0|Mt-0|Oy-0|Po-0	ETS
1861	T	G	Edi-0		ETS

1907	G	T		Tsu-0	ETS
1923	C	G	Col-0|Kn-0|Mt-0	Ct-1	ETS
1931	C	Ins	Ct-1|Kn-0|Wu-0		ETS
1935	T	C		No-0	ETS
1950	T	C		Edi-0|Mt-0|Oy-0|Po-0	ETS
1961	A	T	Can-0		ETS
1975	A	T	Ct-1		ETS
2017	T	C	Ct-1|Hi-0		ETS
2020	G	C		Zu-0	ETS
2032	T	ref	Kn-0|Oy-0|Rsch-4	Col-0|Ler-0|Po-0	ETS
2037	C	T		Ler-0	ETS
2043	C	T		Bur-0	ETS
2104	G	A	Kn-0		ETS
2105	G	A		Bur-0	ETS
2129	C	T	Can-0		ETS
2147	A	G	Can-0		ETS
2154	T	C	Ler-0	Hi-0|Zu-0|Ler-0	ETS
2157	A	T	Ct-1		ETS
2166	G	T		Hi-0	ETS
2168	C	T		Bur-0	ETS
2323	T	Ins	Oy-0		18S
2393	A	G	Sf-2		18S
2591	C	G	Wil-2	Wil-2	18S
2849	G	A		Bur-0	18S
2882	T	C	Ct-1|No-0|Ws-0		18S
2892	G	A		Rsch-4	18S
3270	T	A	Ler-0		18S
3477	C	T		Bur-0	18S
4028	C	A	Ct-1		ITS
4033	G	A		Bur-0	ITS

4080	T	C	Can-0		ITS
4088	C	T	No-0		ITS
4094	C	T	Can-0		ITS
4116	A	G	Kn-0|Ct-1	Edi-0|Rsch-4|Sf-2|Tsu-0|Ct-1	ITS
4155	T	C		Bur-0	ITS
4180	G	A		Wil-2	ITS
4246	G	Ins	Hi-0		ITS
4256	C	ref	Kn-0|Oy-0|Rsch-4	Col-0|Ler-0|Po-0	ITS
4442	T	Ins	Mt-0	Edi-0|Oy-0	ITS
4444	C	T	Ler-0		ITS
4449	C	Ins	Col-0|No-0|Ws-0|Ler-0	Rsch-4|Sf-2|Tsu-0|Ler-0	ITS
4465	C	T	No-0|Ws-0		ITS
4466	G	A	No-0		ITS
4478	G	A		Ct-1	ITS
4531	T	C		Oy-0	ITS
4594	A	ref	Kn-0|Oy-0|Rsch-4	Col-0|Ler-0|Po-0	ITS
4616	C	A		No-0	ITS
4755	C	T		Bur-0	25S
4851	G	T		Wu-0	25S
4855	G	A		Ws-0	25S
4863	T	C	Wu-0		25S
5083	C	T		Po-0	25S
5126	G	A		Wil-2	25S
5178	T	C		Ct-1	25S
6010	G	A		Bur-0	25S
6033	G	T	Ler-0	Ler-0	25S
6046	G	A	Oy-0		25S
6164	C	A	Oy-0		25S
6211	G	A		Wil-2	25S
6235	G	A	Can-0		25S

6254	T	G	Wil-2		25S
6282	G	A		Po-0	25S
6286	G	A		Ws-0	25S
6301	A	T	Can-0		25S
6506	T	C	Sf-2		25S
6661	G	A		Bur-0	25S
6663	C	T		Po-0	25S
6674	C	T		Bur-0	25S
6783	T	A		Edi-0	25S
7624	C	T	Wu-0		25S
7871	G	A	Ct-1		25S
7885	T	C	Kn-0		25S

ETS: External transcribed spacer.

ITS: Internal transcribed spacer.

Table S2. NOR-specific markers in the parental accessions of the F_2 population: Algutsrum and TDr-9.

Position	Reference	Alternative	NOR2	NOR4	Annotation
405	A	ref	TDr-9	TDr-9	ETS
901	C	ref	TDr-9	TDr-9	ETS
1417	C	G		TDr-9	ETS
1683	T	C		TDr-9	ETS
1861	T	G	TDr-9		ETS
1906	C	ref	TDr-9	TDr-9	ETS
1917	C	ref	TDr-9	TDr-9	ETS
1918	A	ref	TDr-9	TDr-9	ETS
2445	T	C		Algutsrum	18S
3904	C	G	Algutsrum		18S
4078	C	Del		TDr-9	ITS
4325	G	A	Algutsrum		5.8S

ETS: External transcribed spacer.

ITS: Internal transcribed spacer.

Table S3. NOR-specific markers in accession Cvi-0.

Position	Reference	Alternative	NOR2	NOR4	Annotation
551	C	T	Cvi-0	Cvi-0	ETS
721	T	C	Cvi-0	Cvi-0	ETS
768	T	C	Cvi-0	Cvi-0	ETS
1109	G	C	Cvi-0	Cvi-0	ETS
1378	G	A		Cvi-0	ETS
1392	A	G	Cvi-0		ETS
1400	A	G	Cvi-0	Cvi-0	ETS
1703	T	G	Cvi-0	Cvi-0	ETS
1902	A	G	Cvi-0	Cvi-0	ETS
4080	T	C	Cvi-0	Cvi-0	ITS
4601	T	C		Cvi-0	ITS
7811	T	C	Cvi-0	Cvi-0	25S
7812	A	C	Cvi-0	Cvi-0	25S

ETS: External transcribed spacer.

ITS: Internal transcribed spacer.
