# Supplementary material for: Epistatic and allelic interactions control expression of ribosomal RNA gene clusters in Arabidopsis thaliana
Source: Genome Biol. 2017 May 3;18:75. doi: 10.1186/s13059-017-1209-z (PMC5414317; doi:10.1186/s13059-017-1209-z)
Supplement: Supplementary file 4 — Nucleolar association of rDNA-2 and rDNA-4 in five accessions. (PDF 55 kb) [file 13059_2017_1209_MOESM4_ESM.pdf]

Additional file 4

Nucleolar association of NOR2 and NOR4 in five accessions. Relative frequency of leaf nuclei with a particular NOR configuration in regard to its close proximity to the nucleolus for the founder accessions Bur-0 (7058), Col-0 (6909), Ct-1 (7067), No-0 (7273) and Sf-2 (7328). n is the number of nuclei in each category.

	**NORs associated with the nucleolus**							
**Accession name** (n= number of nuclei)	**NOR2 2x** n (%)	**NOR2 1x** n (%)	**NOR2 2x & NOR4 1x** n (%)	**NOR2 2x & NOR4 2x** n (%)	**NOR2 1x & NOR4 1x** n (%)	**NOR2 1x & NOR4 2x** n (%)	**NOR4 1x** n (%)	**NOR4 2x** n (%)
Bur-0 (n = 34)	0	0	0	2 (6%)	7 (21%)	6 (18%)	7 (21%)	12 (35%)
Col-0 (n = 33)	0	0	0	12 (36%)	1 (3%)	11 (33%)	1 (3%)	8 (24%)
Ct-1 (n = 49)	20 (41%)	1 (2%)	19 (39%)	9 (18%)	0	0	0	0
No-0 (n = 39)	5 (13%)	2 (5%)	19 (49%)	11 (28%)	2 (5%)	0	0	0
Sf-2 (n = 28)	0	0	0	4 (14%)	4 (14%)	12 (43%)	5 (18%)	3 (11%)
