# Supplementary material for: Epistatic and allelic interactions control expression of ribosomal RNA gene clusters in Arabidopsis thaliana
Source: Genome Biol. 2017 May 3;18:75. doi: 10.1186/s13059-017-1209-z (PMC5414317; doi:10.1186/s13059-017-1209-z)
Supplement: Supplementary file 5 — Figure S1. Different accessions express either rDNA-2 or rDNA-4, or both. Figure S2. The pattern of rRNA expression is consistent across replicate lines. Figure S3. Expression of Bur-0 rDNA-4 when used as a mother in an F1 cross. Figure S4. Cvi-0 expresses rDNA-4. Figure S5. Col-0 rDNA-2 is not reactivated in mutants of the RdDM pathway. (PDF 991 kb) [file 13059_2017_1209_MOESM5_ESM.pdf]

Additional file 5

Figure S1. Different accessions express either rDNA-2 or rDNA-4, or both. The mean proportion of RNA-seq reads expressing a particular reporter variant (y-axis) against the proportion of DNA-seq reads accounting for the existence of the same variant (x-axis) for five natural inbred lines: Edi-0 (7111), Ws-0 (7396), Wu-0 (7415), Can-0 (7063), Hi-0 (8304), Ler-0 (7213) and Zu-0 (7417). Error bars represent standard deviations of 3 biological replicates. Notice that no variants Can-0 rDNA-4-specific passed the threshold (present in > 5% of copies within an individual) presumably due to the small size of that rDNA cluster. The dashed line indicates the one-to-one ratio between DNA and RNA.

Figure S2. The pattern of rRNA expression is consistent across replicate lines. (**A**) The proportion of RNA-seq reads expressing a particular reporter variant (y-axis) against the proportion of DNA-seq reads accounting for the existence of the same variant (x-axis) for MAGIC lines 18, 303 and 319. These MAGIC lines inherited the same genotypes at both rDNA loci after undergoing independent pedigrees. (**B**) Similar to (A), but for a different combination of genotypes at rDNA loci in MAGIC lines 81 and 117. (**C**) Similar to (A), but for MAGIC lines 67 and 188 that inherited both rDNA clusters from founder accession Col-0. (**D**) Similar to (C), but for MAGIC lines 96 and 120 that inherited both rDNA clusters from founder accession No-0. For all subfigures, error bars represent standard deviations of 2 biological replicates, and the dashed line indicates the one-to-one ratio between DNA and RNA.

Figure S3. Expression of Bur-0 rDNA-4 when used as a mother in an F_1 cross. (**A**) The mean proportion of RNA-seq reads expressing a particular reporter variant (y-axis) against the proportion of DNA-seq reads accounting for the existence of the same variant (x-axis) for F_1: ♀ Col-0 x ♂ Sf-2. (**B**) Similar to (A), but for F_1: ♀ Bur-0 x ♂ Col-0. Contrary to the reciprocal cross ♀ Col-0 x ♂ Bur-0, we detected expression of Bur-0 rDNA-4. This discrepancy suggests that the onset of nucleolar dominance may be impacted by a maternal control.

Figure S4. Cvi-0 expresses rDNA-4. The mean proportion of RNA-seq reads expressing a particular reporter variant (y-axis) against the proportion of DNA-seq reads accounting for the existence of the same variant (x-axis) for accession Cvi-0 (6911). Error bars represent standard deviations of five biological replicates. The dashed line indicates the one-to-one ratio between DNA and RNA. For both subfigures, error bars represent standard deviations of 4 biological replicates. The dashed line indicates the one-to-one ratio between DNA and RNA.

Figure S5. Col-0 rDNA-2 is not reactivated in mutants of the RdDM pathway. RT-PCR analysis of rRNA gene 3' variants — VAR1-4, first described by Pontvianne *et al.* (2010) — in WT Col-0 (lanes 2-4), *dcl2/3/4* triple (lanes 5-7) and *nrpd1a-3* single (lanes 8-10) mutant plants. Recently, Chandrasekhara *et al.*, (2016) mapped VAR1 to rDNA-2 in Col-0. Amplification of *Actin-2* mRNA (*ACT2*) was used as a control of total amount of RNA in each sample. Genomic DNA for all assays was loaded in lane 1. RT-PCR without the reverse transcriptase gave no PCR product (-RT panels).

Figure S1

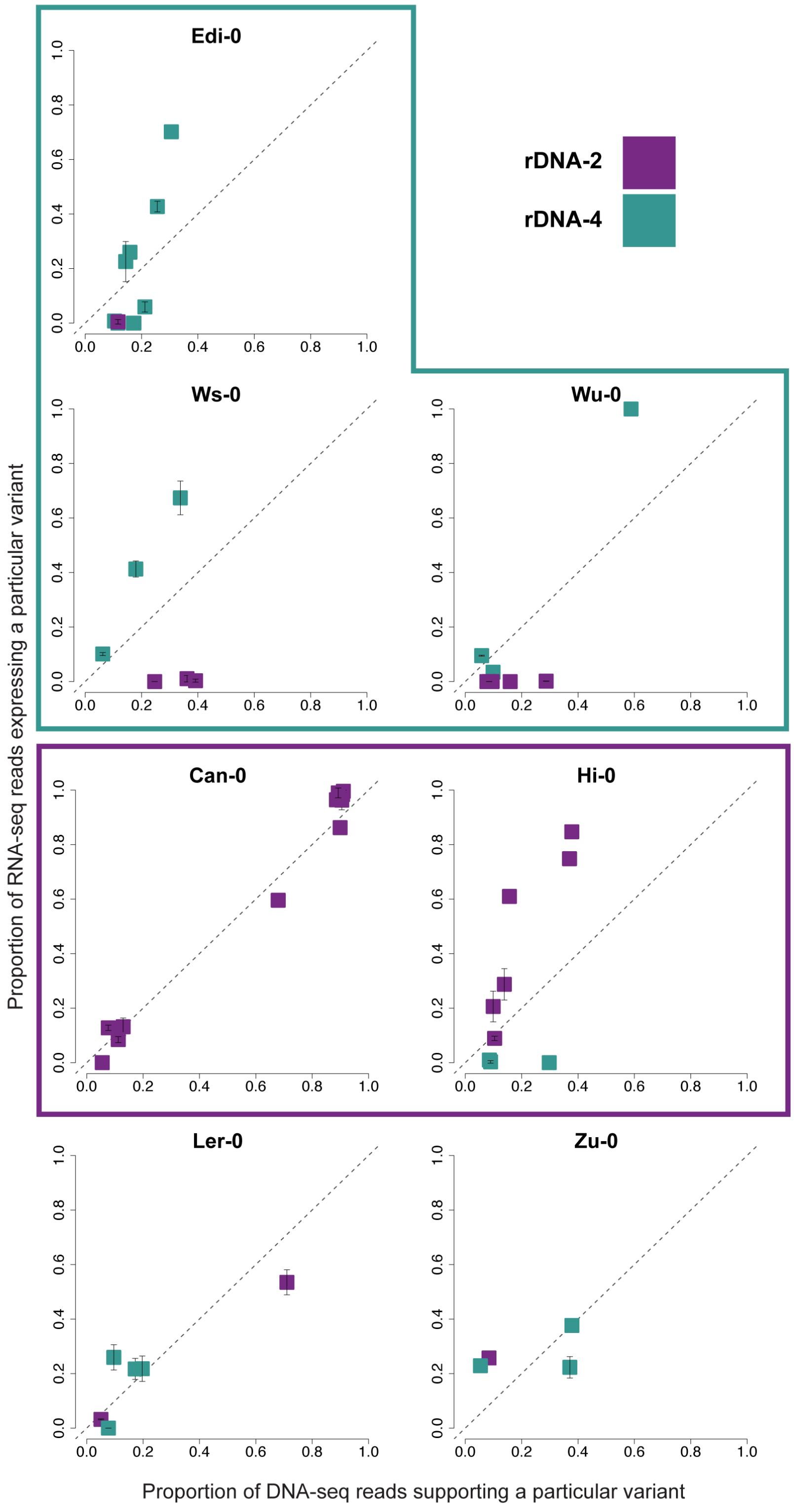
Edi-0
Ws-0
Wu-0
Can-0
Hi-0
Ler-0
Zu-0
rDNA-2
rDNA-4
0.0
0.2
0.4
0.6
0.8
1.0
Proportion of RNA-seq reads expressing a particular variant
Proportion of DNA-seq reads supporting a particular variant

Figure S2

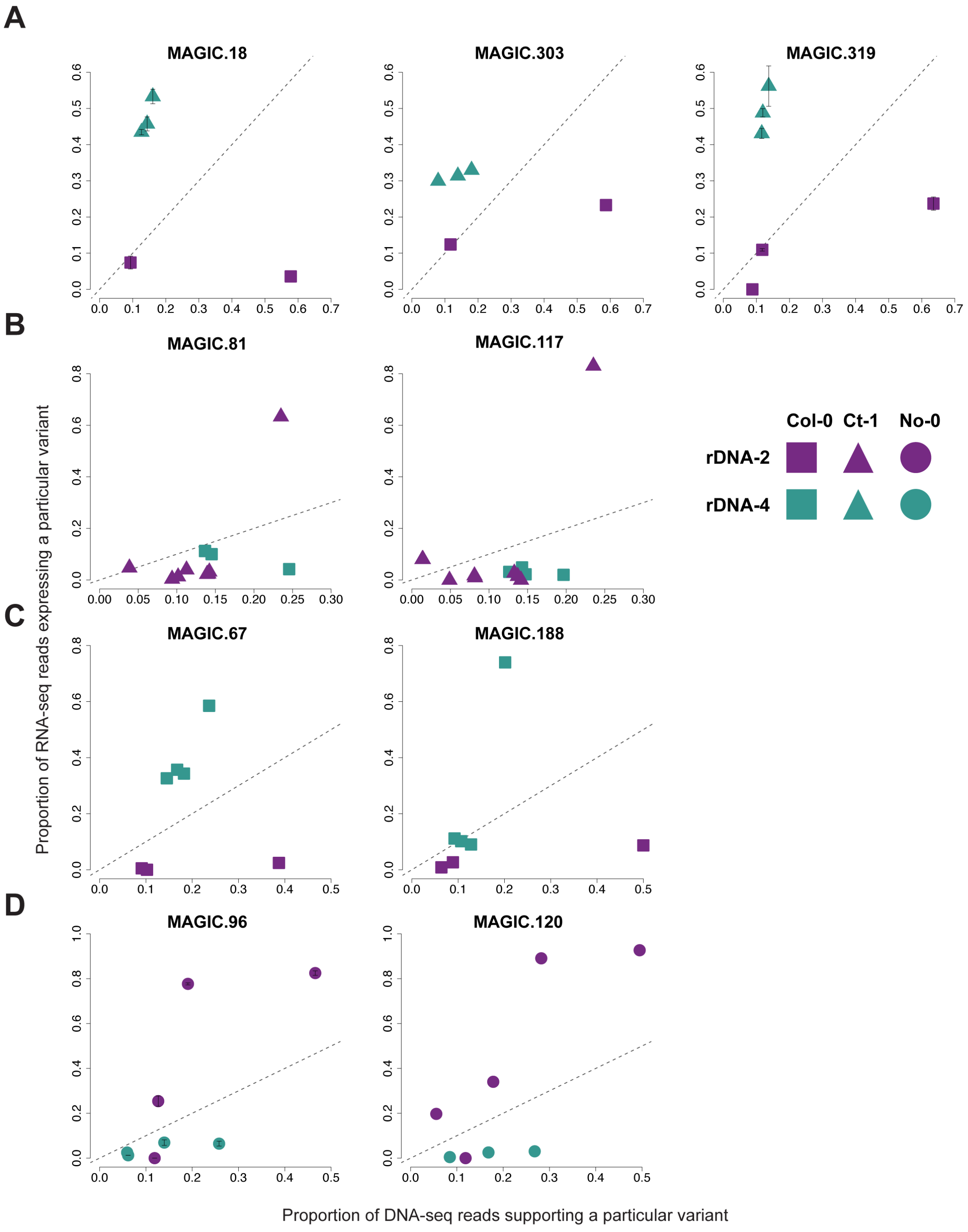
A
MAGIC.18
MAGIC.303
MAGIC.319
B
MAGIC.81
MAGIC.117
C
MAGIC.67
MAGIC.188
D
MAGIC.96
MAGIC.120
Col-0 Ct-1 No-0
rDNA-2
rDNA-4
Proportion of RNA-seq reads expressing a particular variant
Proportion of DNA-seq reads supporting a particular variant

Figure S3

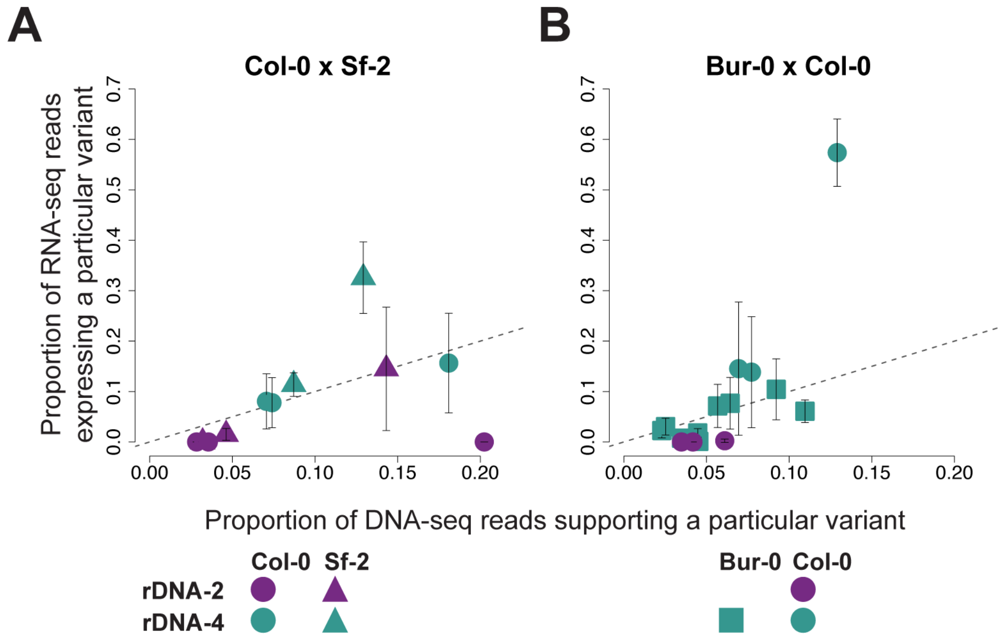
A
B
Col-0 x Sf-2
Bur-0 x Col-0
Proportion of RNA-seq reads expressing a particular variant
0.0
0.1
0.2
0.3
0.4
0.5
0.6
0.7
0.00
0.05
0.10
0.15
0.20
Proportion of DNA-seq reads supporting a particular variant
Col-0 Sf-2
rDNA-2
rDNA-4
Bur-0 Col-0

Figure S4

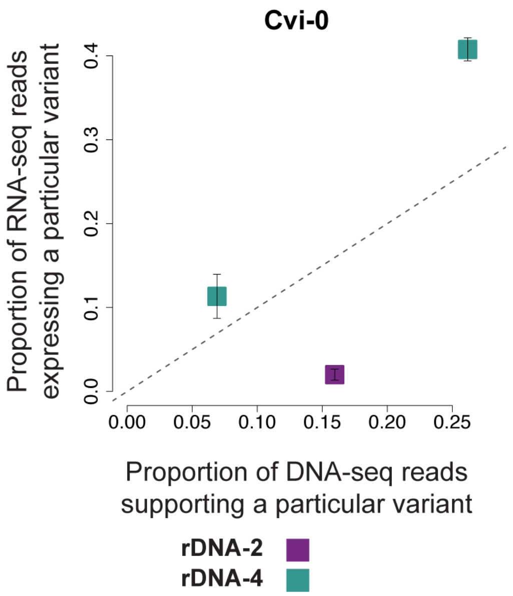

Cvi-0
Proportion of RNA-seq reads
expressing a particular variant
0.0
0.1
0.2
0.3
0.4
0.00
0.05
0.10
0.15
0.20
0.25
Proportion of DNA-seq reads
supporting a particular variant
rDNA-2
rDNA-4

Figure S5

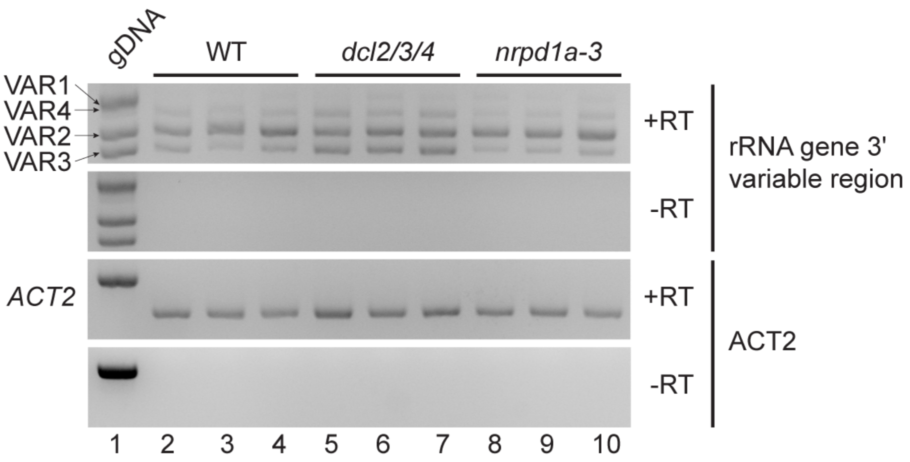
gDNA
WT
dcl2/3/4
nrpd1a-3
VAR1
VAR4
VAR2
VAR3
+RT
-RT
rRNA gene 3' variable region
ACT2
+RT
-RT
ACT2
1 2 3 4 5 6 7 8 9 10
