# Supplementary material for: Epistatic and allelic interactions control expression of ribosomal RNA gene clusters in Arabidopsis thaliana
Source: Genome Biol. 2017 May 3;18:75. doi: 10.1186/s13059-017-1209-z (PMC5414317; doi:10.1186/s13059-017-1209-z)
Supplement: Supplementary file 8 — Data availability. (PDF 69.2 kb) [file 13059_2017_1209_MOESM8_ESM.pdf]

Additional file 8

Data availability.

Data set	No. of samples[†]	Library description	DNA or RNA	Accession number	Reads mapping to 45S rRNA gene	Figures	Reference
The 1001 Genomes Consortium	1,134	N/A	DNA	SRP056687	N/A	N/A	(1001 Genomes Consortium 2016)
Founders of the MAGIC population	3	N/A	DNA	ERP000565	N/A	N/A	(Gan et al. 2011)
Accession Col-0	1	N/A	DNA	SRS597867	N/A	N/A	(Watson et al. 2016)
MAGIC lines	393	N/A	DNA	PRJEB19252	N/A	N/A	(Imprialou et al. 2017)
F_2 population	16	N/A	DNA	PRJNA380541	N/A	N/A	This study
F_1 crosses	10	N/A	DNA	PRJNA380541	N/A	N/A	This study
RIL population	8	N/A	DNA	PRJNA326502	N/A	N/A	(Rabanal et al. 2017)
Founders of the MAGIC population	61	SE 82 bp	RNA	GSE30720<br>GSE30795<br>GSE53197	7.5%	2A, S1	(Gan et al. 2011)
F_2 population	183	SE 50 bp	RNA	GSE92568	9.7%	5B-D, 5F	This study
F_2 population and parents	172	SE 100 bp	RNA*	PRJNA380541	60.7%	5A, 5E	This study
F_1 individuals	20	SE 100 bp	RNA*	PRJNA380541	63.1%	4, S3	This study
MAGIC lines	18	PE 100 bp	RNA	GSE94107<br>GSE96812	16.8%	3, S2	(Imprialou et al. 2017)
Accession Cvi-0	4	PE 100 bp	RNA	ERR754070<br>ERR754076<br>ERR754056	16.7%	S4	(Clauw et al. 2015)

				ERR754055			
Accession Cvi-0	1	SE 100 bp	RNA	GSM2135874	6.1%	S4	(Kawakatsu et al. 2016)

† Includes replicates if available.

* Total RNA-seq library.

N/A. Not applicable.
